# Daytime and nighttime heatwave intensity and acute care utilization for mental and neurological disorders in California

**DOI:** 10.1088/2752-5309/ae3128

**Published:** 2026-01-06

**Authors:** Yiqun Ma, Kristen Guirguis, Caitlin G Jones-Ngo, Anais Teyton, Haley E Brown, Fiona Charlson, Michael Jerrett, Rachel Connolly, Alexander Gershunov, Miriam E Marlier, Tarik Benmarhnia

**Affiliations:** 1 Scripps Institution of Oceanography, University of California San Diego, La Jolla, CA, United States of America; 2 Herbert Wertheim School of Public Health and Human Longevity Science, University of California San Diego, La Jolla, CA, United States of America; 3 School of Public Health, San Diego State University, San Diego, CA, United States of America; 4 School of Public Health, Faculty of Medicine, University of Queensland, Herston, Australia; 5 Queensland Centre for Mental Health Research, Queensland Health, Wacol, Australia; 6 Department of Environmental Health Sciences, Fielding School of Public Health, University of California Los Angeles, Los Angeles, CA, United States of America; 7 Luskin Center for Innovation, University of California Los Angeles, Los Angeles, CA, United States of America; 8 Irset Institut de Recherche en Santé, Environnement et Travail, UMR-S 1085, Inserm, University of Rennes, EHESP, Rennes, France

**Keywords:** heatwave, mental health, neurological disorder, acute care utilization, California

## Abstract

Heatwave exposures have been linked to a variety of mental and neurological disorders. Little is known, however, about the potentially differential associations of daytime versus nighttime heatwave intensity with subtypes of mental and neurological disorders. In this time-stratified case-crossover study, we estimated and compared the associations of typically dry daytime and typically humid nighttime heatwave intensities, characterized by heatwave indices (HWIs), with acute care utilizations for various subtypes of mental and neurological disorders in 1412 ZIP Code Tabulation Areas in California from 2006 to 2019. A total of 4309 294 acute care utilizations for mental disorders and 2097 563 for neurological disorders were included in this study. Higher associations with nighttime HWI were found for most disease subtypes, including anxiety disorder, depressive disorder, schizophrenia, bipolar disorder, post-traumatic stress disorder, Alzheimer’s disease and related dementias, and Parkinson’s disease; while daytime HWI showed a higher impact on conduct disorders (*P* < .001). On average, during the warm season in California, nighttime heatwaves accounted for about 70.6% and 34.0% of acute care utilizations for mental and neurological disorders that were attributable to heatwaves, respectively. Our findings highlight the detrimental impacts of humid nighttime heatwaves on mental and neurological health and call for innovative heat preparedness actions and increased awareness among public health practitioners as more nighttime heatwaves are anticipated under climate change.

## 1. Introduction

Heatwaves are increasingly recognized as an important risk factor for mental and neurological health. In recent years, a growing number of studies have linked extreme heat and heatwaves to symptom exacerbation and increased hospitalization risks for mental or neurological disorders, such as anxiety disorders, schizophrenia, bipolar disorder, depression, headaches, Alzheimer’s disease and related dementias (ADRD), and Parkinson’s disease [1–9]. Heat exposure challenges the human body’s capacity to maintain a narrow temperature range and can directly affect molecular functions, particularly that of ion channels [2]. Factors such as reduced thermoregulatory capacity, medication use, maladaptive behaviors, and social isolation make individuals with mental or neurological conditions more susceptible to heat-related adverse health outcomes as well [10, 11].

Rising global atmospheric moisture levels have amplified humid heat events that result in muggy heat persisting through the night, i.e. nighttime heat events [12, 13]. Higher humidity exacerbates heat stress because it reduces the efficiency of sweat evaporation [14]. Daytime and nighttime heatwaves are typically driven by distinct meteorological mechanisms related to humidity, cloud cover, and other thermodynamic processes [12, 15]. Specifically, in California, the heatwave activities in warm season have been categorized into two distinct categories: (1) typically dry daytime heatwaves (hereafter ‘daytime-accentuated’ or ‘daytime’ heatwaves, interchangeably); and (2) humid nighttime-accentuated heatwaves, which result when daytime heat does not cool down much at night due to high humidity (hereafter ‘nighttime-accentuated’ or ‘nighttime’ heatwaves, interchangeably) [16]. This classification is related to, but distinct from, another commonly used scheme of daytime-only, nighttime-only, and day-night compound heatwaves [15]. In our framework, day-night compound heatwaves are classified as either daytime-accentuated or nighttime-accentuated, depending on the period during which excess heat is more dominant [16].

Despite these distinct meteorological patterns, most studies on the association between heatwaves and mental or neurological disorders either only focused on maximum temperature, i.e. daytime expression of heatwaves, or did not differentiate daytime and nighttime heatwaves [1, 8, 9]. Daytime and nighttime heatwaves, however, may have differential health impacts due to the influence of distinct local meteorological conditions [12, 15], reduced overnight recovery from heat stress [17–19], and the potential for nighttime heat to disrupt sleep and exacerbate mental and neurological conditions [10, 20]. Compared to daytime heatwaves, nighttime heatwaves may pose a greater threat to human health, because high and sustained temperatures during the night reduce people’s ability to cool down and recover from daytime heat exposure, thereby increasing the risk of heat-related illnesses and deaths [17–19]. This is further exacerbated by the urban heat island effect, which can lead to higher minimum temperatures in urban areas due to the slow radiative cooling from urban structures, providing less nighttime relief [21]. In addition, high nighttime temperatures have been shown to disturb normal sleep rhythm, shorten sleep duration, and reduce sleep quality [20], thereby worsening mental and neurological conditions [10]. To the best of our knowledge, however, no epidemiological studies have been conducted to systematically compare the associations of daytime versus nighttime heatwave exposures with various specific mental and neurological disorders.

In addition, existing evidence suggests that heat-related health impacts are not evenly distributed across populations. Studies in diverse settings have reported differential heat-related health risks, including mental health outcomes, by sex, age, race and ethnicity, and regions, although the identified vulnerable populations often differ by health outcome and context [4, 5, 8, 22]. However, it remains unclear whether and how these sociodemographic and regional disparities differ for daytime versus nighttime heatwaves in relation to mental and neurological disorders.

The intensity of heatwaves is also important. Most previous studies have defined heatwaves as a binary exposure, comparing health outcomes on heatwave versus non-heatwave days [23]. While this is a classical and widely used approach, it does not account for potential differences in health impacts across varying heatwave intensities. In California, researchers have developed daytime and nighttime heatwave indices (HWIs) to quantify the intensity of heatwaves during the day and night [16]. These indices allow us to examine exposure-response relationships between heatwave intensity and mental and neurological outcomes for effects of both daytime and nighttime heatwaves.

Evidence indicates a greater increase in nighttime heatwaves (compared to daytime) in the U.S., both in past observations and future projections [16, 24–26]. Thus, given the potentially higher impacts of nighttime heatwaves, accurately quantifying the health effects of each heatwave type separately is critical to avoid underestimating the overall heatwave-attributable burden of mental and neurological disorders. Furthermore, while the United Kingdom and several European countries explicitly incorporate both daytime and nighttime temperatures into their heat warning systems, many heat action plans and interventions in the U.S., such as heat alerts and cooling centers, are primarily triggered by daytime conditions and often operate only during daytime hours, providing limited coverage during hot nights [27, 28]. A better understanding of nighttime heatwave impacts on mental and neurological disorders could enable policymakers, caregivers, and healthcare practitioners to improve protection for patients with these conditions.

Therefore, utilizing statewide daily acute care utilization data in California from May to October, 2006–2019, this study focuses on three aims: (1) investigate and compare the associations between daytime and nighttime heatwave intensities and acute care utilizations for overall mental and neurological disorders, as well as for a wide range of specific mental and neurological diseases; (2) examine how these associations vary by sex, age, race and ethnicity, and subregions; and (3) quantify the acute care utilization burden attributable to dry daytime versus humid nighttime heatwaves and compare their contributions to each specific mental and neurological condition in California.

## 2. Methods

This time-stratified case-crossover study utilized air temperature and acute care utilization data from May to October, 2006–2019 for 1412 ZIP Code Tabulation Areas (ZCTAs) in California with a population of at least 1000. This study was approved by the California Health and Human Services Agency’s Committee for the Protection of Human Subjects (project number: 2021–116).

### 2.1. Acute care utilization for mental and neurological disorders

We obtained data on daily unscheduled hospital and emergency department visits in California, collectively referred to as ‘acute care utilizations’, from the California Department of Health Care Access and Information [29]. This dataset includes each patient’s residential ZIP code, date of visit, sex, age, race and ethnicity, and primary International Classification of Diseases (ICD)-9 or ICD-10 code.

This study covered acute care utilizations from two major categories: mental and behavioral disorders (hereafter ‘mental disorders’; ICD-9: 290–319; ICD-10: F00–F99) and diseases of the nervous system (hereafter ‘neurological disorders’; ICD-9: 320–389; ICD-10: G00–G99). Within mental disorders, we specifically examined acute care utilizations for anxiety disorder, depressive disorder, schizophrenia, bipolar disorder, conduct disorder, and post-traumatic stress disorder (PTSD). Within neurological disorders, specifically we evaluated headaches and Parkinson’s disease. We also included ADRD, which span both mental and neurological diagnostic categories. The corresponding ICD-9 and ICD-10 codes used to identify these specific diseases were listed in supplementary methods 1. We matched each ZIP code to its corresponding ZCTA and aggregated the individual-level data into daily ZCTA-level acute care utilization counts for each type of disease.

### 2.2. Daytime and nighttime heatwave indices

The calculation of daytime and nighttime HWIs was adapted from a previously published method [16]. Daily minimum and maximum air temperature (*T*_min_ and *T*_max_) data from 1981 to 2019 were obtained from the gridded meteorological reanalysis product, which is a spatially and temporally complete, high-resolution (4 km × 4 km) gridded dataset of surface meteorological variables for the contiguous United States [30]. For each grid cell, we calculated the 95th percentile of the daily *T*_max_ and *T*_min_ during May–October over the climate reference period (1981–2010) as local thresholds. On each day, the degrees by which *T*_max_ and *T*_min_ exceeded their respective thresholds were calculated (set to zero if below the threshold). A heatwave was defined as at least two consecutive days in which either *T*_max_ or *T*_min_ exceeded the local threshold. A daytime-accentuated heatwave was classified when the sum of excess *T*_max_ during the heatwave was higher than the sum of excess *T*_min_, and vice versa for nighttime-accentuated heatwaves [16]. The daily excess *T*_max_ and *T*_min_ during a heatwave are daytime and nighttime HWIs, representing the intensity of daytime and nighttime heatwaves. HWIs were coded 0 on non-heatwave days. We extracted the daily ZCTA-level daytime and nighttime HWIs using the population-weighted centroid of each ZCTA.

### 2.3. Statistical analysis

We used a time-stratified case-crossover design to estimate associations between daytime and nighttime HWIs and acute care utilizations for mental and neurological disorders. This study design, in which each case-day is compared to other comparable control-days, has been widely applied to examine the acute health effects of short-term environmental exposure [4, 31–34]. In our study, a case day was defined as the date of acute care utilization, and the matched control days were identified as those on the same day of the week from other weeks within the same calendar month and year, in the same ZCTA. This widely used time-stratified self-matching strategy controlled for confounding variables that remain relatively stable within a month, including individual-level factors such as sex, age, race and ethnicity, income, education, as well as any time-invariant behavior factors, and ZCTA-level characteristics such as socioeconomic status, population density, and built environment [33, 34]. The main model with conditional quasi-Poisson regression can be expressed as

\begin{equation*}{\text{Log}}\left( {E\left( Y \right)} \right) = \alpha + {\beta _1}{\text{DHWI}} + {\beta _2}{\text{NHWI}} + {\beta _3}{\text{Stratum}}\end{equation*}

where $Y$ denotes daily count of cause-specific acute care utilizations; ${\text{DHWI}}$ and ${\text{NHWI}}$ represent daily daytime and nighttime HWIs, respectively; ${\text{Stratum}}$ is the indicator for matched case and control days; and $\alpha $ is the intercept. We reported percent change in acute care utilization associated with each 1 °C increase in daytime or nighttime heatwave exposure, which was calculated by $\left( {\exp \left( {{\beta _1}{\text{ or }}{\beta _2}} \right) - 1} \right) \times 100\% $. In a secondary analysis, we used binary definitions of daytime and nighttime heatwaves. We replaced ${\text{DHWI}}$ and ${\text{NHWI}}$ with ${\text{DHW}}$ and ${\text{NHW}}$ in the model, which are binary indicators of each event. To explore the potential non-linearity of the association between HWIs and health outcomes, we also used a natural cubic spline to model daytime and nighttime HWIs, with 2 internal knots positioned at the 10th and 50th percentiles of the HWI distribution. This modeling choice is consistent with a previous study [35].

Subgroup analyses were performed to explore the potential effect modification by sex (male and female), age (0–14, 15–64, and 65 and above), and race & ethnicity (non-Hispanic White, non-Hispanic Black, non-Hispanic Asian, and Hispanic). In secondary analyses, we further stratified age into finer categories (0–14, 15–24, 25–44, 45–64, and 65 and above) and considered subgroups defined by intersections among sex, age, and race & ethnicity. We also conducted stratified analysis by subregion, comparing coastal ZCTAs (defined as ZCTAs with population-weighted centroids within 30 miles of the Pacific coastline) versus inland ZCTAs. To compare model estimates between subgroups or strata, if the estimates are ${E_1}$ and ${E_2}$ with standard errors (SEs) ${\text{S}}{{\text{E}}_{\text{1}}}$ and ${\text{S}}{{\text{E}}_{\text{2}}}{\text{,}}$ then the difference $d = {\text{ }}{E_1} - {\text{ }}{E_2}{\text{ }}$ has ${\text{S}}{{\text{E}}_d} = { }\sqrt {{\text{SE}}_1^2 + {\text{SE}}_2^2} $ [36]. Statistical significance was evaluated at *P*  < 0.05 (two-sided test). To account for multiple comparisons in subgroup analysis, Bonferroni correction was performed when comparing subgroups. In addition, we explored the lag pattern in the associations from the current day (lag 0), up to 6 d after exposure (lag 6).

Based on the estimated coefficients of daytime and nighttime HWIs (${\beta _1}$ and ${\beta _2}$), we further calculated the attributable number (AN) of acute care utilizations for each mental and neurological disorder using an attributable fraction (AF) method. For each disease and HWI type, ${\text{AF}} = 1 - {{\text{e}}^{ - \beta {\text{HWI}}}}$ and ${\text{AN}} = {\text{AF}} \times {\text{Total acute care utilizations}}$. This calculation was performed each day in each ZCTA, and then the total AN was summed to annual state-level estimates.

We conducted several sensitivity analyses to test the robustness of our results: (1) we used the ZCTA-specific 90th percentile of historical temperatures as the heatwave threshold; (2) we used 3 or 4 consecutive days to define heatwaves; (3) we restricted the study period to 2006–2014, which only used ICD-9 codes; and (4) we adjusted for relative humidity in the model. All statistical analyses were conducted with R software (version 4.3.1; R Development Core Team) using the package *gnm*. R code for this analysis is available at https://github.com/benmarhnia-lab/HW_mental_neurological.

## 3. Results

### 3.1. Descriptive statistics

**Figure 1.**
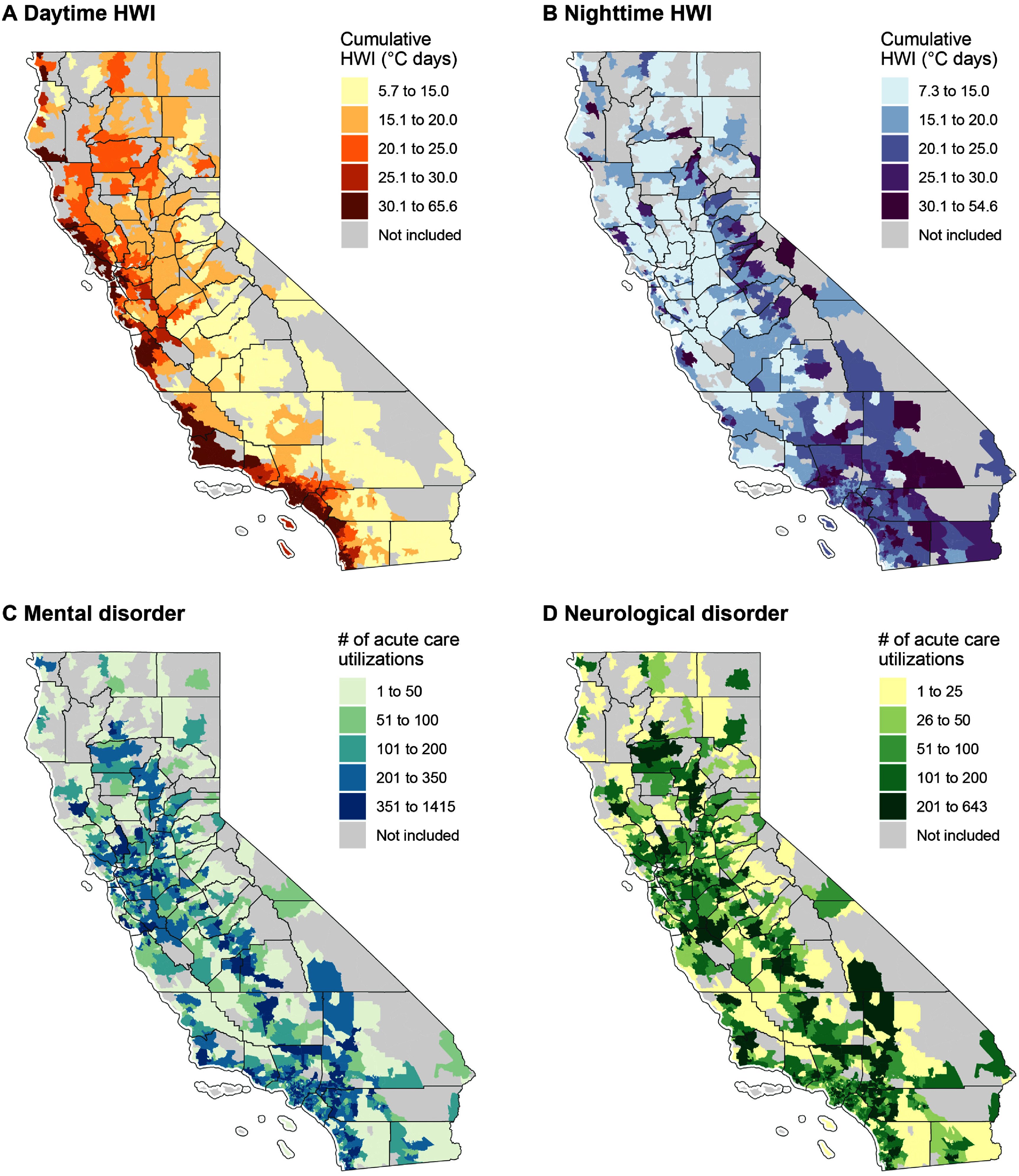
Spatial distribution of cumulative daytime and nighttime heatwave indices and acute care utilizations for mental and neurological disorders. These maps display the average annual cumulative daytime- (A) and nighttime-accentuated (B) heatwave indices (HWIs) and annual average number of acute care utilizations for mental (C) and neurological (D) disorders in each ZCTA. Grey areas indicate ZCTAs with a population of less than 1,000 which were not included in the study.

Across 1412 ZCTAs in California from 2006 to 2019, each ZCTA experienced an average of 9 (standard deviation (SD): 4) daytime-accentuated heatwave days and 8 (SD: 2) nighttime-accentuated heatwave days each year, May–October. The average annual cumulative daytime HWI was 26 °C days and the average annual cumulative nighttime HWI was 19 °C days per year (SD: 12 and 7, respectively) (table 1). In general, the cumulative daytime HWI was higher in coastal regions (figure 1(A)), whereas the cumulative nighttime HWI was higher in inland areas (figure 1(B)). Both daytime and nighttime HWIs showed great variations across years (supplementary figure 1).

**Table 1.** Descriptive statistics of annual heatwave days, heatwave indices, and mental or neurological acute care utilizations per ZCTA (May–October).

	Mean (SD)	P5	P25	Median	P75	P95
*Average number of heatwave days per ZCTA per year*						

Daytime heatwave	9 (4)	4	6	9	12	17
Nighttime heatwave	8 (4)	2	4	8	11	17

*Average cumulative HWI per ZCTA per year (°C days)* ^a^						

Daytime HWI	26 (12)	12	17	22	34	50
Nighttime HWI	19 (7)	10	13	19	24	31

*Average number of acute care utilizations per ZCTA per year* ^b^						

Mental disorder	218 (204)	9	51	168	329	620
Male	115 (115)	4	25	84	168	344
Female	103 (92)	4	25	82	156	274
Aged 0–14	9 (9)	0	2	6	13	26
Aged 15–64	191 (183)	7	44	143	284	553
Aged 65 and above	18 (16)	1	4	15	27	46
Non-Hispanic White	107 (99)	5	28	82	157	298
Non-Hispanic Black	25 (52)	0	1	6	24	122
Non-Hispanic Asian	9 (13)	0	0	4	11	38
Hispanic	63 (89)	1	6	27	83	257
Neurological disorder	106 (98)	5	28	81	157	303
Male	41 (38)	2	11	31	61	115
Female	65 (61)	3	17	48	94	190
Aged 0–14	6 (6)	0	1	4	8	19
Aged 15–64	83 (81)	4	20	61	124	246
Aged 65 and above	17 (14)	1	5	15	25	42
Non-Hispanic White	51 (50)	3	14	37	71	144
Non-Hispanic Black	13 (29)	0	0	3	11	68
Non-Hispanic Asian	4 (7)	0	0	2	5	18
Hispanic	33 (46)	0	3	14	43	135
Anxiety disorder	38 (36)	1	9	28	55	109
Depressive disorder	33 (30)	1	7	26	50	90
Schizophrenia	25 (36)	0	3	12	32	96
Bipolar disorder	16 (15)	0	3	12	24	47
Conduct disorder	2 (2)	0	0	1	2	6
PTSD	1 (1)	0	0	0	1	2
Headache	55 (51)	2	13	40	80	158
ADRD	3 (3)	0	1	3	5	9
Parkinson’s disease	1 (1)	0	0	1	1	2

ZCTA, ZIP Code Tabulation Areas; HWI: heatwave index; SD, standard deviation; P5, 5th percentile; P25, 25th percentile; P50, 50th percentile; P75, 75th percentile; P95, 95th percentile; PTSD: post-traumatic stress disorder; ADRD: Alzheimer’s disease and related dementias.

^a^ Mean annual sum of HWI values across heatwave days in each ZCTA, averaged over the study years.

^b^ A detailed description of the subgroups for each specific disease is presented in supplementary table 1.

A total of 4309 294 acute care utilizations for mental disorders and 2097 563 acute care utilizations for neurological disorders were included in this study. Their spatial distributions are shown in figures 1(C) and (D). Overall, acute care utilizations for both mental and neurological disorders increased over the study period (supplementary figure 2). The average annual number of acute care utilizations per ZCTA was 218 (SD: 204) for mental disorders and 106 (SD: 98) for neurological disorders. Descriptive information for specific disease types and subgroups is provided in tables 1 and supplementary table 1.

### 3.2. Exposure-response relationship between HWI and acute care utilization

**Figure 2.**
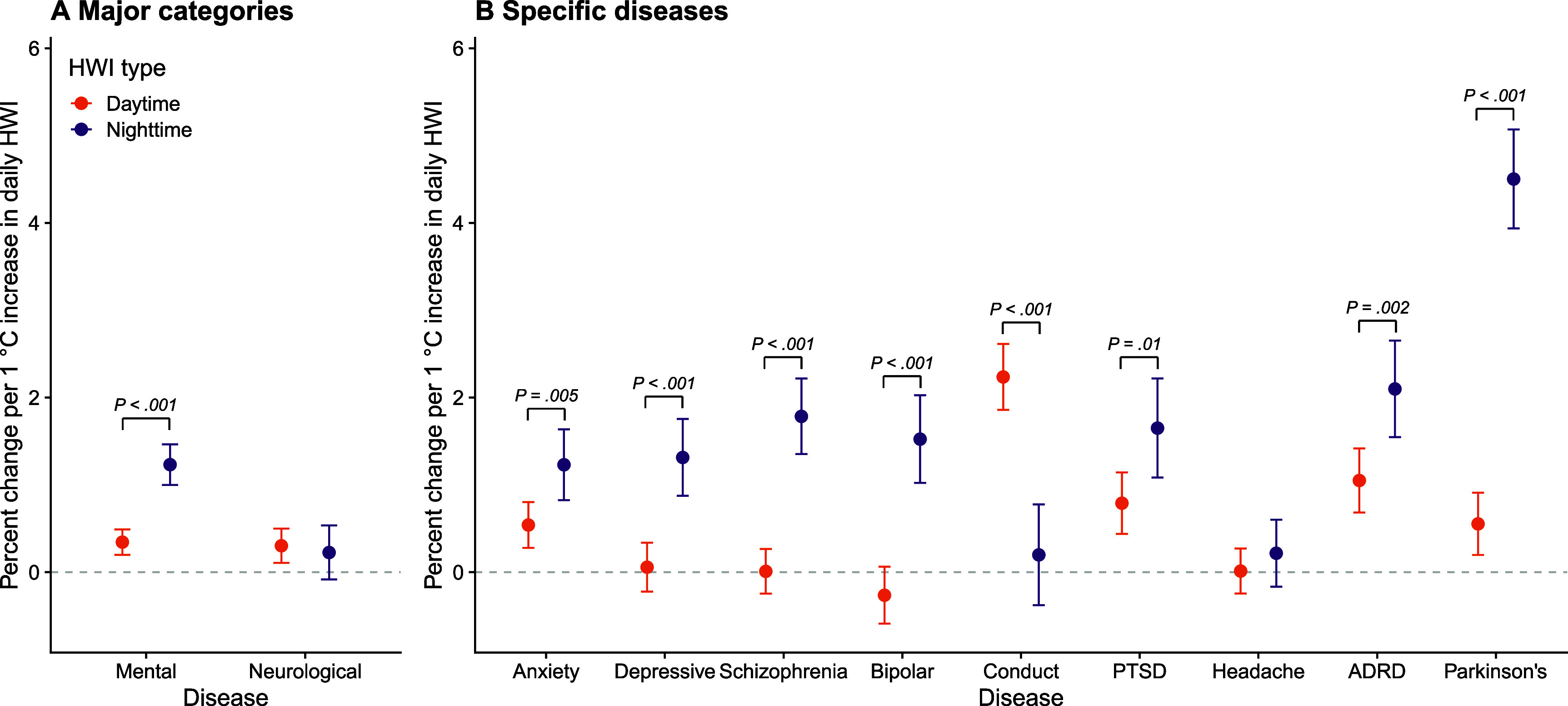
Association between daytime and nighttime heatwave indices and acute care utilizations for mental and neurological disorders. This figure presents the percent change in daily mental and neurological disorder acute care utilization risk per 1 ˚C increase in daily daytime or nighttime heatwave index (HWI). Panel A shows the results for the two major categories; panel B shows the results for specific diseases (mental disorders: anxiety disorder, depressive disorder, schizophrenia, bipolar disorder, conduct disorder, and post-traumatic stress disorder [PTSD]; neurological disorders: headaches and Parkinson’s disease; Alzheimer’s disease and related dementias [ADRD] span both categories). The error bars represent 95% confidence intervals. The *P* values from heterogeneity tests indicate the statistical significance of the differences in estimates between daytime and nighttime HWIs.

Daytime and nighttime HWIs were positively associated with increased acute care utilization risk for mental disorders, with nighttime HWI showing a larger effect (figure 2(A)). Specifically, a 1 °C increase in daily nighttime HWI was associated with a 1.23% (95% confidence interval (CI): 1.00%, 1.46%) increase in acute care utilization for mental disorders, which was 0.88 (95% CI: 0.61, 1.16) percentage points significantly higher than the daytime estimates (0.34% [95% CI: 0.20%, 0.49%]; *P*< .001 [*P* for heterogeneity]). No significant difference between daytime (0.30% [95% CI: 0.11%, 0.50%]) and nighttime (0.23% [95% CI: −0.08%, 0.53%]) HWI was observed for overall neurological acute care utilizations (*P* = .68) (figure 2(A); supplementary table 2).

The exposure-response relationships for specific mental and neurological disorders are shown in figure 2(B). Compared to daytime HWIs, increases in nighttime HWIs showed stronger associations for anxiety disorder (*P* = .005), depressive disorder (*P* < .001), schizophrenia (*P* < .001), bipolar disorder (*P* < .001), PTSD (*P* = .01), ADRD (*P* = .002), and Parkinson’s disease (*P* < .001). For conduct disorder, however, the association was higher for daytime HWI (*P* < .001). For headache, no significant association was observed for either daytime or nighttime HWIs (figure 2(B); supplementary table 2).

When using a traditional binary heatwave definition, no significant difference between daytime and nighttime heatwave events was observed for both mental and neurological disorders. A significantly higher effect estimate was found for conduct disorder (*P* < .001), PTSD (*P* = .01) and Parkinson’s disease (*P* < .001), which is likely due to the overall higher intensity of daytime heatwaves (supplementary figure 3; tables 1).

### 3.3. Results of subgroup and stratified analyses

**Figure 3.**
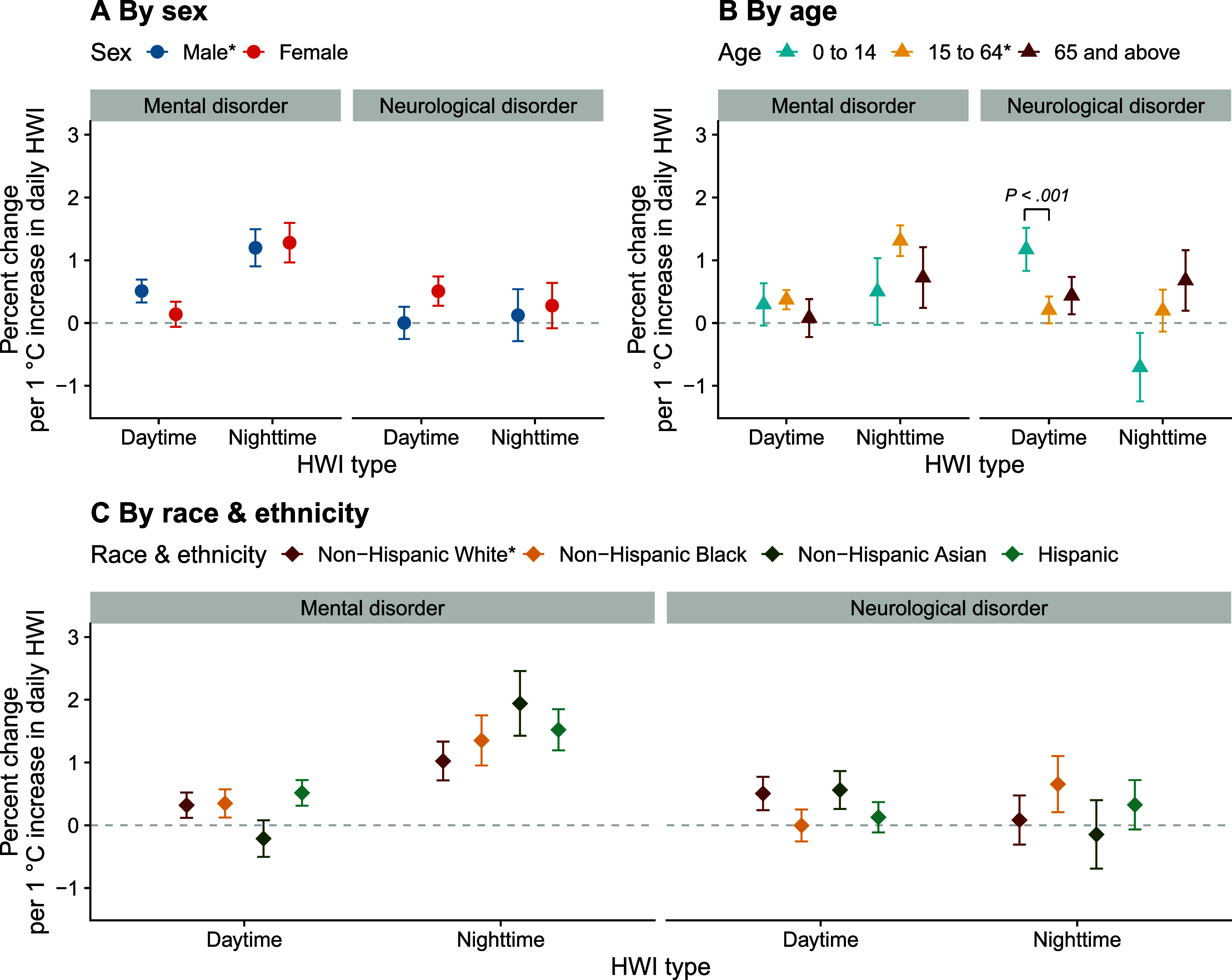
Association between daytime and nighttime heatwave indices and acute care utilizations for mental and neurological disorders in population subgroups. This figure presents the percent change in daily mental and neurological disorder acute care utilization risk per 1 ˚C increase in daily daytime or nighttime heatwave index (HWI), specific to each population subgroup. The error bars represent 95% confidence intervals. The *P* values from heterogeneity tests indicate the statistical significance of the differences in these estimates between groups (after Bonferroni correction). The asterisk (*) indicates the subgroup that was used as the reference.

We examined the association between daytime and nighttime HWIs and acute care utilizations for mental and neurological disorders across different sex, age, and race and ethnicity groups (figure 3; supplementary table 3). For both mental and neurological disorders, there was no significant difference in effect estimates between males and females, regardless of daytime or nighttime HWIs. In the subgroup analysis by age, the effect estimate for neurological disorder acute care utilizations was higher among those aged 0–14 for daytime HWI, using the 15–64 group as the reference (*P* < .001). This pattern remained when we applied finer age categories and used the 25–44 group as the reference (supplementary figure 4). Based on the results from subgroups defined by intersections of sex and age and of age and race & ethnicity, this higher vulnerability among children aged 0–14 years appeared to be driven primarily by girls and by Asian children in this age group (supplementary figure 5). Among different race & ethnicity groups higher central estimates were found for the associations between nighttime HWIs and acute care utilizations for mental disorders among non-Hispanic Black, Asian and Hispanic people compared to non-Hispanic White people, but the difference was not statistically significant after Bonferroni correction.

**Figure 4.**
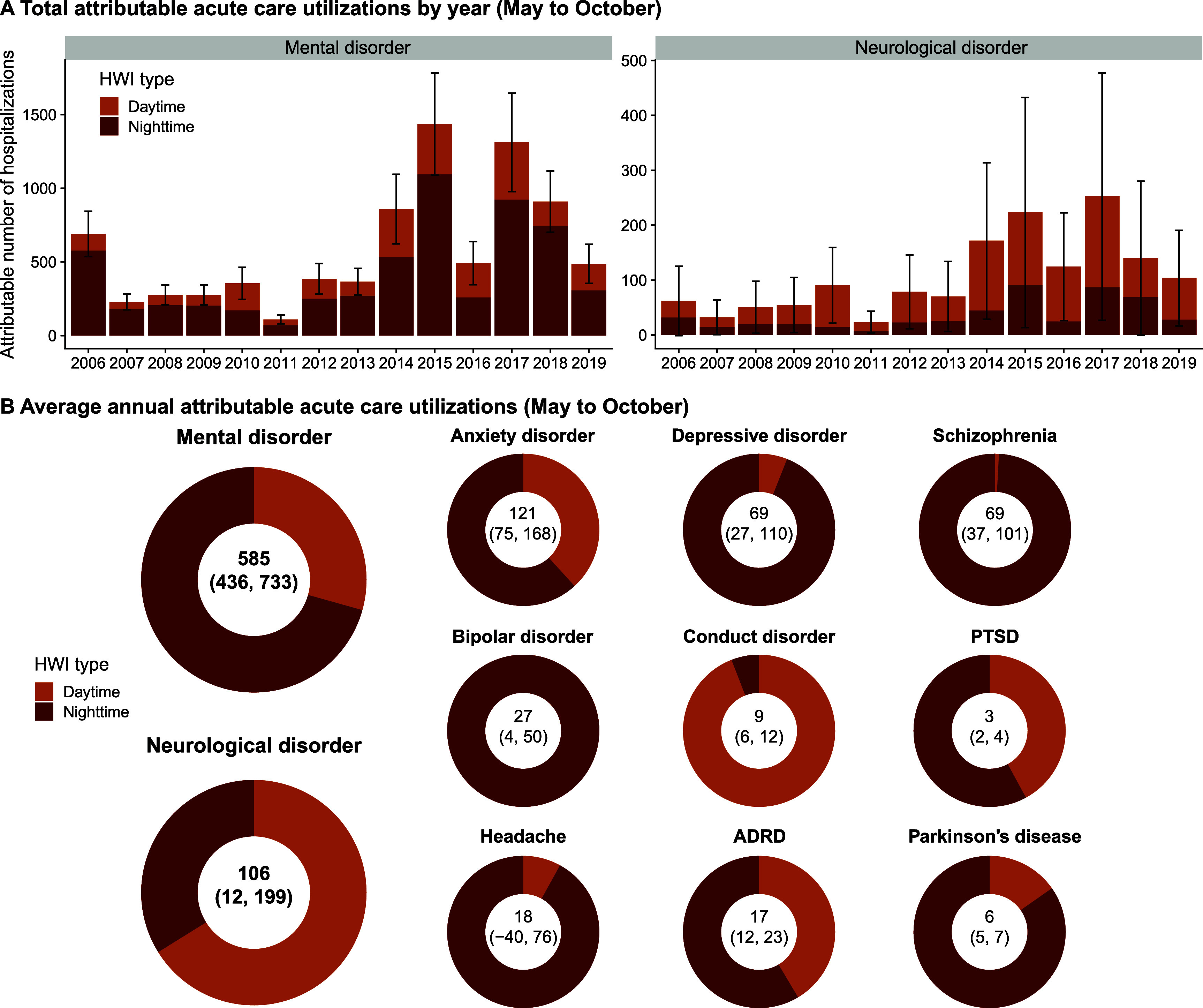
Acute care utilizations for mental and neurological disorders attributable to daytime and nighttime heatwave exposures. This figure above presents the annual number of acute care utilizations attributable to heatwave exposures in each year over the study period, calculated based on daytime and nighttime heatwave indices (HWIs, May to October, 2006-2019) (A). The error bars represent 95% confidence intervals (CIs) of the sum of estimated number of excess acute care utilizations attributable to both heatwave types. The figure below shows the average annual attributable acute care utilizations for each disease (May to October) (B). The sum of excess hospitalizations attributable to both heatwave types and the 95% CIs were noted in the center of each ring.

In stratified analyses by region, increases in nighttime HWI were associated with a greater increase in acute care utilizations for mental disorders in coastal areas compared to inland areas. No notable regional differences were observed for neurological disorders (supplementary figure 6).

### 3.4. Attributable acute care utilization burden

Figure 4(A) shows the annual number of acute care utilizations for mental and neurological disorders attributable to daytime and nighttime-accentuated heatwaves, calculated based on the cumulative intensity of heatwaves and the exposure-response relationship between HWIs and acute care utilization risks. Overall, the total number of acute care utilizations attributable to heatwaves increased over the study period, with substantial inter-annual variations. Nighttime heatwaves contributed a greater fraction for mental disorders and daytime heatwaves contributed more to neurological disorders. On average, approximately 585 (95% CI: 436, 733) excess acute care utilizations for mental disorders were attributable to heatwaves in California per year, of which about 70.6% were contributed by nighttime heatwaves. For neurological disorders, approximately 106 (95% CI: 12, 199) annual excess acute care utilizations were attributable to both types of heatwaves, with nighttime heatwaves accounting for about 34.0% of that burden (figure 4(B)). Similarly, most of the studied specific diseases had more acute care utilizations attributable to nighttime heatwaves, except for conduct disorder, which was predominantly contributed by daytime heatwaves. Detailed ANs and fractions for each disease are provided in supplementary table 4.

### 3.5. Results of sensitivity analysis

Our main findings generally remained robust under different methodological variations (supplementary figure 7). Regarding lag effects, increases in both daytime and nighttime HWIs were associated with increased acute care utilizations for mental disorders for up to 4 or 5 d. For neurological disorders, no notable lag effect was observed (supplementary figure 8). In addition, we explored potential non-linear relationships between HWIs and acute care utilizations for mental and neurological disorders. The estimated exposure-response curves suggested an approximately linear association within the range where most HWI values were observed (supplementary figure 9).

## 4. Discussion

This study presents some of the first results revealing the differential associations between daytime and nighttime heatwave intensities and acute care utilizations for a wide range of specific mental and neurological diseases. While increases in intensity of both types of heatwaves were associated with increased risk of acute care utilization for mental disorders, increases in nighttime heatwave intensity had a stronger effect. No significant difference between daytime and nighttime HWIs was observed for overall neurological acute care utilizations; however, higher associations with nighttime HWI were found for ADRD and Parkinson’s disease. Each year, we estimated that more than 580 acute care utilizations for mental disorders and over 100 for neurological disorders that are attributable to heatwaves in California in the warm season, with nighttime heatwaves accounting for about 71% and 34% of these attributable acute care utilizations, respectively. These findings build on the broader literature on heatwaves and mental and neurological health and highlight the importance of mitigation and adaptation measures targeting nighttime heatwaves to protect patients with mental and neurological disorders.

Our finding that nighttime heatwaves have a greater impact than daytime heatwaves on acute care utilizations for overall mental disorders and a few specific neurological diseases is consistent with previous studies. For example, a recent study in China focusing on the overall category of mental disorders found that although both hot days and hot nights were associated with increased outpatient visits, the effect of hot nights was greater than that of hot days [37]. Another study using a Chinese nationwide cohort of middle-aged and older adults reported stronger associations of depressive symptoms with nighttime and compound heatwaves than with daytime heatwaves [38]. However, neither of them examined the intensity of hot days and nights or the intensity of heatwaves. This observed higher risk associated with nighttime-accentuated heatwave intensity could be explained by reduced nocturnal cooling and recovery from daytime heat and disturbed sleep quality and duration [19, 20].

For conduct disorder, the effects of daytime heatwave intensity were greater than those of nighttime heatwaves. This result aligns with a recent U.S. cohort study which reported an association between extreme heat (characterized by maximum temperatures) and a small increase in externalizing symptoms in preadolescence and early adolescence [39]. Although existing evidence on the relationship between heat and conduct disorder is limited, one possible explanation is that high daytime temperatures may disrupt daily routines by restricting outdoor activities, potentially leading to frustration, aggression, and an increased likelihood of oppositional or defiant behaviors [40], especially among children and adolescents.

We estimated that daytime heatwaves accounted for a larger proportion of neurological disorder-related acute care utilizations than nighttime heatwaves; however, this difference was not statistically significant and primarily reflects the combination of a slightly higher central estimate in the exposure-response relationship for daytime HWI and greater cumulative daytime HWI over the study period. Whether daytime heatwaves are intrinsically more harmful for neurological disorders remains unclear both in this study and in existing literature and warrants further investigation in future studies.

Compared to people aged 15–64, children aged 0–14 were found to be more vulnerable to increases in daytime heatwave intensity, with higher acute care utilization risks for neurological disorders (figure 3(B)). This finding aligns with the overall literature on heat and pediatric health [41], although research on heat and children’s neurological health is still emerging [39]. A multicenter study in the U.S. found that extreme heat was associated with higher risks of emergency department visits for nervous system diseases among children [42]. Compared to adults, children are particularly susceptible to heat-induced adverse health outcomes due to their underdeveloped thermoregulation, a higher risk of dehydration, and the inability to implement common measures to reduce ambient temperature [43]. The higher association observed for daytime heatwave intensity in this age group may partly reflect exposure patterns: daytime heatwaves coincide with outdoor play and sports, when children are more likely to be physically active and directly exposed to ambient heat and sunlight, whereas at night most children are indoors and their sleeping environments are actively managed by parents or caregivers [44]. In addition, the estimated effect of daytime heatwave intensity was more pronounced for neurological than for mental disorders in this age group, but the underlying mechanisms are not well understood and need further physiological and epidemiological investigation.

We found suggestive evidence that increases in nighttime heatwave intensity were associated with a greater rise in acute care utilizations for mental disorders in coastal areas compared to inland regions. This finding is concerning because coastal heatwaves in California are projected to intensify even relative to the background warming induced by climate change [16, 45, 46]. Given the region’s high population density and relatively low acclimatization to heat, particularly humid heat, the combination of more frequent and intense nighttime heatwaves and stronger exposure-response relationships may lead to a disproportionate mental health burden in coastal California communities in the future.

Heatwaves, especially nighttime heatwaves, contributed to a substantial burden of mental disorders and specific neurological diseases. The findings of our study could inform the psychiatric and neurological societies in updating clinical practice guidelines to incorporate extreme heat, especially nighttime heatwaves, into the care of patients with mental and neurological conditions. In line with recent viewpoints from clinicians, service providers could routinely assess heat vulnerability (e.g. living alone, limited access to cooling, use of medications that impair thermoregulation) and provide anticipatory counseling before the warm season; assisted living and long-term care facilities could also set appropriate standards for cooling capacity, backup power, and staff training, and active monitoring of residents with mental and neurological disorders during both daytime and nighttime heat events [47, 48]. In addition, to enhance community resilience to heatwaves, policymakers should account for nighttime heatwaves when designing heat warning system and action plans, and ensure sufficient resources that are accessible to people with mental and neurological conditions. For example, local agencies could incorporate nighttime heatwave indices into alert thresholds, extend cooling interventions (e.g. cooling centers) into the night, and ensure that advisories and warnings effectively reach people with mental and neurological disorders and their caregivers [27, 28]. Climate change is increasing the frequency, intensity, and duration of heatwaves globally, particularly in California, where rising humidity has contributed to more frequent and intense nighttime heatwaves [24, 25]. In this context, local agencies and healthcare providers should be prepared to meet the rising demand for mental and neurological health services.

Some limitations of this study should be noted. First, although we examined a range of specific mental and neurological disorders, we did not explore heterogeneity within each condition (e.g. different types of headaches) due to sample size considerations. Second, our study period included both ICD-9 and ICD-10 coding systems, which may introduce inconsistencies in disease classification. Yet, our main results remained robust in a sensitivity analysis restricted to the ICD-9 period. Third, due to data limitations, we were unable to explore the potentially differential vulnerabilities by individual-level factors such as occupation, medication use, air conditioning availability, and social isolation [49, 50]. In addition, potential effect modification by community-level characteristics, such as socioeconomic factors, and their roles in driving spatial heterogeneity in vulnerability, warrant further investigation. Finally, Santa Ana winds, which are dry gusty winds, often cause coastal heatwaves in fall, winter and spring in California [51]. Such heatwaves may have different health impacts compared to heatwaves during warm seasons. Future research should further investigate the differential impacts of daytime and nighttime heatwaves on more specific mental and neurological conditions, extend the analyses to other seasons, explore the physiological and social pathways underlying these associations, and examine a broader range of effect modifiers.

## 5. Conclusion

While increased intensities of both daytime and nighttime heatwaves were associated with increased acute care utilization risk for mental and neurological disorders, nighttime heatwave intensity had a larger impact on mental disorders and several subtypes of neurological disorders. As nighttime heatwaves are expected to become more frequent and intense under climate change, the burden of healthcare utilization among patients with these conditions is likely to increase. Our findings therefore call for integrating nighttime temperatures into the design of heat action plans, extending key adaptation measures into nighttime hours where feasible, and raising awareness among public health practitioners to better protect patients with mental and neurological disorders in a warming climate.

## Data Availability

The data that support the findings of this study are openly available at the following URL/DOI: www.climatologylab.org/gridmet.html/; https://hcai.ca.gov/ [29, 30]. Supplementary data available at https://doi.org/10.1088/2752-5309/ae3128/data1.
